# Long-term clinical outcome and phenotypic variability in hyperphosphatemic familial tumoral calcinosis and hyperphosphatemic hyperostosis syndrome caused by a novel *GALNT3* mutation; case report and review of the literature

**DOI:** 10.1186/s12863-014-0098-3

**Published:** 2014-09-24

**Authors:** Silje Rafaelsen, Stefan Johansson, Helge Ræder, Robert Bjerknes

**Affiliations:** Department of Clinical Science, University of Bergen, Bergen, Norway; Center of Medical Genetics and Molecular Medicine, Haukeland University Hospital, Bergen, Norway; Department of Pediatrics, Haukeland University Hospital, Bergen, Norway

**Keywords:** Familial hyperphosphatemia, Hyperphosphatemic familial tumoral calcinosis, Hyperphosphatemia hyperostosis syndrome, GALNT3, FGF23

## Abstract

**Background:**

Hyperphosphatemic Familial Tumoral Calcinosis (HFTC) and Hyperphosphatemic Hyperostosis Syndrome (HHS) are associated with autosomal recessive mutations in three different genes, *FGF23*, *GALNT3* and *KL*, leading to reduced levels of fibroblast growth factor 23 (FGF23) and subsequent clinical effects.

**Results:**

We describe a consanguineous family with two affected siblings with HFTC and HHS caused by a novel homozygous G-to T substitution in exon 3 of *GALNT3* (*c.767 G > T*; p.Gly256Val), demonstrating great phenotypic variation and long asymptomatic intervals. Calcific tumors appeared at 14 years of age in the male, and the female displayed episodic diaphysitis from age 9 years. Symptoms of eye involvement were present in both from childhood, and progressed into band keratopathy in the female. Abnormal dental roots and tooth loss, as well as myalgia were present in both from their mid-twenties, while the female also had calcifications in the placenta, the iliac vessels and thyroid cartilage. New calcific tumors appeared more than 20 years after the initial episodes, delaying diagnosis and treatment until the ages of 37 and 50 years, respectively. Both siblings had elevated serum phosphate levels, inappropriately elevated tubular maximum phosphate reabsorption per unit glomerular filtration rate (TmP/GFR), reduced levels of intact FGF23 and increased levels of c-terminal FGF23. Review of all 54 previously published cases of *GALNT3, FGF23,* and *KL* associated HFTC and HHS demonstrated that more subjects than previously recognized have a combined phenotype.

**Conclusion:**

We have described HFTC and HHS in a consanguineous Caucasian family with a novel *GALNT3* mutation, demonstrating new phenotypic features and significant variability in the natural course of the disease. A review of the literature, show that more subjects than previously recognized have a combined phenotype of HFTC and HHS. HHS and HFTC are two distinct phenotypes in a spectrum of *GALNT3* mutation related calcification disorders, where the additional factors determining the phenotypic expression, are yet to be clarified.

**Electronic supplementary material:**

The online version of this article (doi:10.1186/s12863-014-0098-3) contains supplementary material, which is available to authorized users.

## Background

The last decade has brought new insight into the molecular and pathophysiological aspects of phosphate metabolism. Previously, the regulation of serum and body phosphate was thought to be merely a consequence of the regulation of calcium levels by parathyroid hormone (PTH) and vitamin D. It is now accepted that the regulation of phosphate is specific, and involves phosphatonins, in particular fibroblast growth factor 23 (FGF23) [1].

Hyperphosphatemic familial tumoral calcinosis (HFTC) is a rare monogenic disorder with disturbances in the hormonal regulation of phosphate levels by FGF23, leading to soft tissue calcifications [2]. Hyperphosphatemic hyperostosis syndrome (HHS) is characterized by hyperphosphatemia and episodes of diaphysitis and cortical hyperostosis visualized on x-rays. This was thought to be a separate entity, rarely occurring together with HFTC, but it has later been shown that the same genes and same mutations are involved in both HHS and HFTC. This has led to the current opinion, that HHS and HFTC are different manifestations of the same genetic defect, and that in some families the same mutation can lead to either phenotype [3-5]. Both conditions are caused by inactivating mutations in either the *FGF23* gene encoding the phosphaturic hormone FGF23 [6,7], or the *GALNT3* gene encoding the UDP-N-acetyl-alpha-D-galactosamine:polypeptide N-acetylgalactosaminyltransferase 3 (GalNAc-T3) enzyme [8]. This enzyme is necessary to glycosylate FGF23, thereby preventing the break down and inactivation of the functional full-length version of the protein [9]. There is also one report of HFTC caused by an inactivating mutation in the *KL* gene, encoding α-Klotho, which is an essential co receptor for the FGF23 receptor function in phosphate regulation [10].

*GALNT3*-associated HHS and HFTC are rarely found in Caucasians [11]. In this report we describe a consanguineous family of Norwegian origin, with a novel homozygous mutation in exon 3 of *GALNT3*, where diagnosis and treatment were delayed until late adulthood. In addition to new phenotypic features, the cases demonstrate the significant variability in the natural course of the disease, and how features of HFTC and HHS can be substantially overlapping with time, even in the same patient.

## Methods

### Patients

The family was recruited from a national population-based cohort of familial hyperphosphatemia in Norway. Written informed consent was obtained from all study participants. The study was approved by the Regional Committee for Medical and Health Research Ethics, Region West, Norway (REK number 2009/1140).

### Biochemical parameters

Blood samples were collected after an overnight fast. Circulating levels of calcium, albumin, phosphate and alkaline phosphatase (ALP) activity in serum were analyzed using the Modular P-system from Roche Diagnostics (Basel, Switzerland). PTH was measured with a two-site chemiluminescent enzyme-labeled immunometric assay for intact PTH (Immulite 2000, Siemens Healthcare Diagnostics, Deerfield, IL, USA). Measurement of 25(OH)D levels was performed using an in-house developed liquid chromatography double mass spectrometry (LC-MS/MS) method [12]. A spot sample of urine collected at the time of blood sampling was analyzed for calcium, phosphorus and creatinine. The maximal tubular reabsorption of phosphate per glomerular filtration rate (TmP/GFR) was calculated according to the algorithm based on the nomogram of Walton and Bijvoet [13,14]. Plasma intact FGF23 (iFGF23) was measured with the FGF23-ELISA kit (Kainos Laboratories, Japan), with a lower detection limit of 3 pg/ml and a coefficient of variation (CV) of 4%. Plasma C-terminal FGF23 (cFGF23) was measured using the FGF23 second generation C-terminal ELISA kit (Immutopics, San Clemente, CA, USA), with a lower detection limit of 1.5 RU/ml and a CV of 4.7%.

### Bone mineral density

The bone mineral density (BMD) of the right hip and lumbar spine was measured with dual X-ray absorptiometry (DXA) using Hologic Delphi W (Hologic inc., Bedford, Massachusetts, USA).

### Genetic analysis

Genomic DNA was purified from blood using the QiaSymphony system (Qiagen, Hilden, Germany).

**Figure 1:**
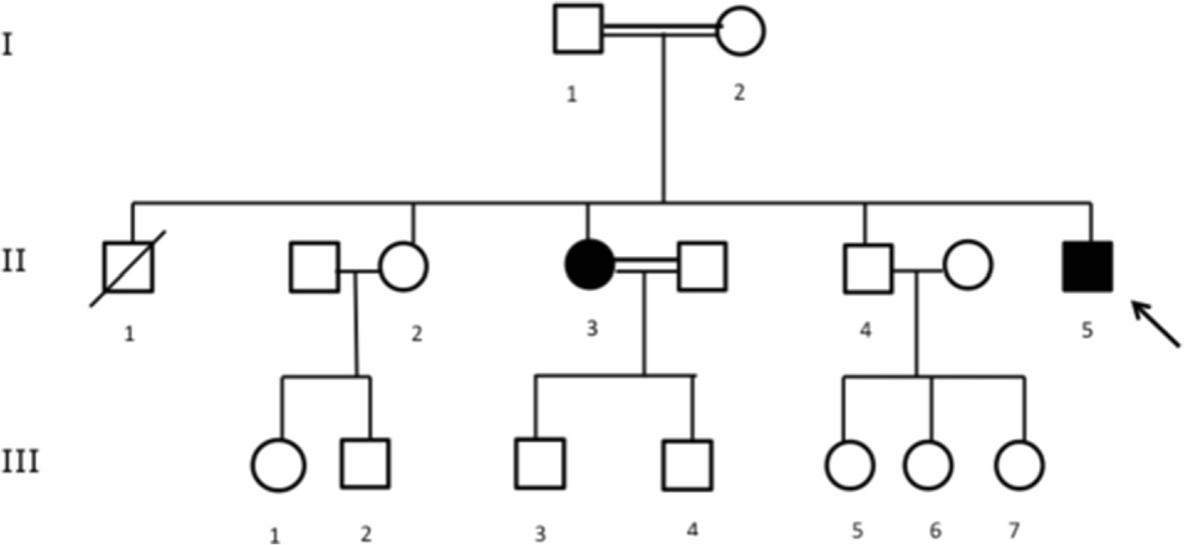
**Pedigree.**

All exons and intron-exon boundaries, of *GALNT3*, *KL* and *FGF23* were sequenced in subject 1 (the index case). Only exon 3 of *GALNT3* was sequenced in subject 2 as well as in the 7 asymptomatic family members (Subjects I-2, II-2, II-4, III-1, III-3, IV-1, IV-2 in Figure 1) and 192 healthy blood donors.

DNA targets were first amplified by polymerase chain reaction (PCR) (list of primers available upon request) using the AmpliTaq Gold® DNA polymerase system (Applied biosystems, Life biosystems, Carlsbad, California, USA. PCR amplicons were purified with 2 μl of ExoSapIT®. Using the Big Dye Terminator® chemistry sequencing was performed on the 3730 DNA analyzer, (Applied biosystems) and analyzed using the SeqScape® software (Applied biosystems).

### Strategy of the literature review

We searched PubMed and EmBase for case reports on HFTC and HHS caused by mutations in *GALNT3*, *FGF23* and *KL* using the search terms “hyperphosphatemic tumoral calcinosis”, “hyperphosphatemia hyperostosis syndrome”, “*GALNT3* mutation” and “*FGF23* mutation” and “*KL* mutation”. Some cases and pedigrees were described in several papers, including earlier papers on the clinical presentation and later papers describing disease progression and genetic diagnose. In these cases we included all papers. We did not include case reports describing pedigrees or cases where a genetic diagnosis was not made.

## Results

### Case reports

**Figure 2:**
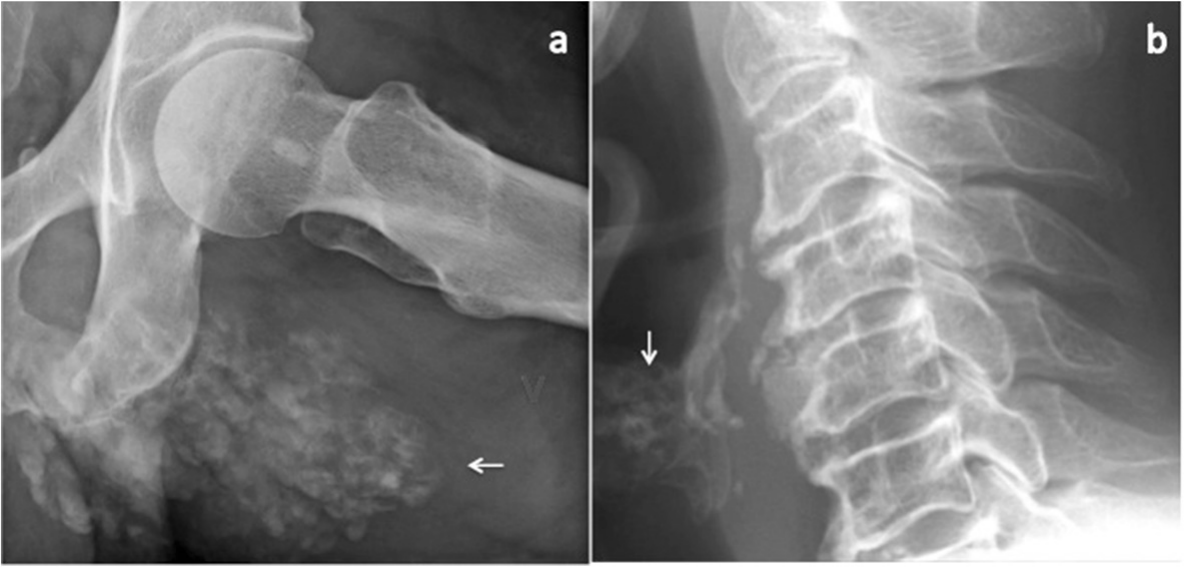
**Radiographic findings.** The pictures show **a)** the left gluteal calcification in subject 1, and **b)** age-inappropriate thyroid cartilage calcifications in subject 2.

*Subject 1* is a Caucasian male, the youngest of five siblings of a consanguineous marriage (Table 1); the parents have a common ancestor eight generations back. He presented with a calcified mass on his right elbow at age 14. This mass was removed, the histological diagnosis was tumoral calcinosis, but no treatment or follow up was initiated. Prior to this episode he had chronic conjunctival irritation and abnormal dental roots shown on x-ray. Dental abscesses and spontaneous tooth loss started at 25 years of age. There were no new calcific tumors until the age of 35, when he developed a tender mass in his left gluteal area. He was eventually diagnosed with HFTC at age 37, when he presented with a 6 × 8 cm calcification over his left ischial tuberosity (Figure 2a), hyperphosphatemia, an inappropriately high TmP/GFR of 1.46 and TRP 84%. His renal function and serum calcium were normal. Serum 1,25 dihydroxyvitamin D3 was inappropriately normal in the setting of hyperphosphatemia and serum intact PTH was low (Table 2). Therapy with the phosphate binder Sevelamer 1600 mg three times daily was started, but the patient discontinued medication after one year due to non-medical circumstances. At age 41 his left gluteal mass had increased further, to the degree that it restricted his daily activity. Spontaneous rupture of the overlying skin resulted in discharge of a white matter and transient pain relief, but there was rapid relapse. Sevelamer 4.8-6.4 g per day was tried once again, but had no effect on tumor size or serum phosphate levels.

**Table 1:** **FGF23 levels**

**Individual** ^**1**^	**Ref. range**	**Unit**	**I-2**	**II-2**	**II-3**	**II-4**	**II-5**	**III-1**	**III-3**
**Genotype** ^**2**^			**M/m**	**M/m**	**m/m**	**M/M**	**m/m**	**M/M**	**M/m**
i-FGF23	21.6-70.2^3^	pg/ml	66.3	51	26.8	33.0	12	47.2	53
c-FGF23	10-80^2^	RU/ml	32.2	25.2	120	34.8	210	17.2	20.2

^1^Individuals, as presented in Figure 1.

^2^M = wild-type allele; m = mutant allele.

^3^Reference range valid for the laboratory used for this study.

**Table 2:** **Biochemical profiles at diagnosis (T1) and inclusion (T2)**

**Parameter**	**Normal range**	**Unit**	**Subject 1**		**Subject 2**	
			**T1**	**T2**	**T1**	**T2**
s-Phosphate	M: 0.85-1.65	mmol/L	1.81	2.21		
	F: 0.85-1.50				1.6 – 1.84	1.36
s-Calcium	2.15-2.51	mmol/L	2.37	2.36	2.5	2.64
s-iPTH	1.6-6.9	pmol/L	0.8	1.4	NA	5.2
s-Creatinine	M: 60-105	μmol/L	72	78		
	F: 45-90				73	61
i-FGF23	26.1-70.2^1^	pg/ml	NA	12	NA	26.8
c-FGF23	10-80^1^	RU/ml	NA	210	NA	120
1,25(OH)_2_ vit D	50-150	nmol/L	85	87	NA	54
TmP/GFR	> 0.85	mmol/L	1.46	2.07	1.41	1.12
TRP	> 80	%	84	94	88	82.5

^1^Reference range valid for the laboratory used for this study.

At 42 years old his left gluteal mass had not decreased despite treatment for one year. He had general myalgia, with pain and stiffness in calves and forearms after slight activity. Clinical examination revealed a large, tender mass in the left gluteal area, displacing the natal cleft to the right, and the calves felt hard on palpation. Eye examination revealed salt-like conjunctival deposits. All his permanent teeth had been substituted by implants, but dental health was otherwise good. His height was 172.2 cm, head circumference was 59 cm and blood pressure was 110/80 mmHg. Computerized tomography of the kidneys and abdomen was normal. Bone mineral density (BMD) of the femoral neck and total hip was relatively high (T-scores 1.3 and 1.9; Z-scores 1.9 and 2.2, respectively), while lumbar spine BMD was normal (T-score 0.4; Z-score 0.5). In conclusion, he has had manifestations of classic HFTC from childhood. The long interval between development of calcific tumors has delayed diagnosis and treatment.

*Subject 2* is the older sister of subject 1. She was diagnosed with HFTC at the age of 50 upon serum phosphate screening of the first-degree relatives of subject 1. A review of her past medical record revealed an episode of several cutaneous tumors on her scalp at three weeks of age. At age six years she had a large tumor in the gluteal area, and at age seven years a large tumor on her right thigh; both tumors were incised, but no diagnose is noted in her medical records. Between the ages of nine and 12 years, she had three episodes of unilateral leg pain lasting for several weeks and treated with antibiotics for suspected osteomyelitis; blood cultures were sterile and x-rays showed sclerosis of the tibia and periosteal thickening with onionskin configuration. At age 25 she had a new episode of leg pain, but this time the radiological examinations were negative, and she received no treatment. From her twenties, she has had conjunctival irritation and dental problems similar to her brother. At age 22 she gave birth to a healthy boy one week before term date after an uneventful pregnancy; the placenta was highly calcified, but the baby had no signs of intrauterine growth restriction (birth weight 3400 g, length 51 cm, head circumference 36 cm). Her second pregnancy was uncomplicated, but birth records are not available. She has had no stillbirths or spontaneous abortions.

In adulthood her chief complaint has been myalgia, stiffness of knees, hips and shoulders, and pain and deformities of her fingers and feet. The clinical findings resembled osteoarthritis, but the rheumatologic diagnostic work up was inconclusive. X-rays showed pronounced calcifications and degenerative changes in and around the phalanges of her hands and feet, calcifications of soft tissues in the foot, large bilateral calcaneal enthesopathies and age-inappropriate calcification of the thyroid cartilage (Figure 2b). Blood tests at age 50 (Table 2) revealed hyperphosphatemia, normal kidney function and inappropriately elevated renal tubular reabsorption of phosphate. The serum level of calcium was slightly elevated, but the serum levels of PTH and 1.25 (OH)_2_ vitamin D_3_ were not available.

At 55 years of age she had been treated with Sevelamer 800 mg per day for the previous three years, and both her symptoms and serum phosphate level had remained stable. Clinical examination revealed a height of 163.3 cm, head circumference was 56 cm and blood pressure was 120/70 mmHg. She had salt-like deposits on her bulbal conjunctiva, and the ophthalmology report confirmed band keratopathy but normal vision. She had some dental implants but good dental health. Findings in her hands, knees and hips were as previously noted. In addition her calves felt stiff on palpation. Computerized urography showed normal kidneys, but calcifications in the iliac vessels. BMDs of the femoral neck, total hip and lumbar spine were all normal (T-scores 1.5, 0.5 and 0.4; Z-scores 2.6, 1.2 and 1.5, respectively). We conclude in retrospect that she first manifested symptoms HHS in childhood, and that the absence of calcific tumors has led to delayed diagnosis.

Biochemical profile at the time of diagnosis and inclusion is given in Table 2.

### FGF23

The plasma intact and c-terminal FGF23 were measured at enrolment. The level of intact FGF23 was decreased and c-terminal FGF23 was elevated in the two affected subjects (Table 1); the deviation from normal was more pronounced in the male than in the female. The unaffected healthy family members had normal levels of iFGF23 and cFGF23 (Table 1).

### Genetic analysis

**Figure 3:**
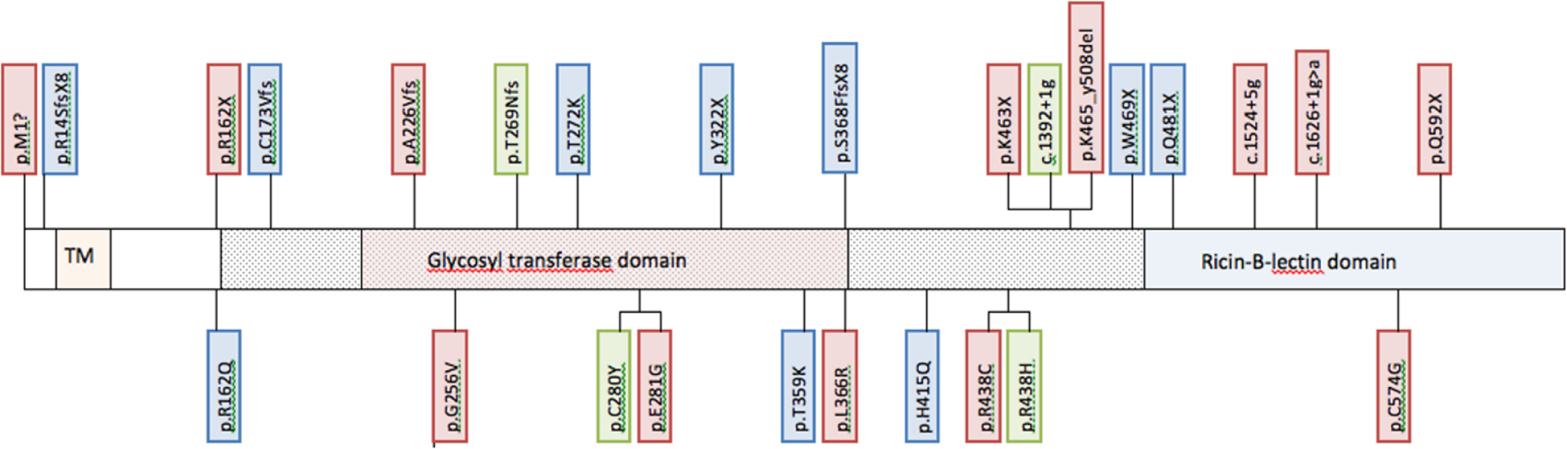
***GALNT3*** **variations [** 3 **,** 4 **,** 8 **,** 11 **,** 18 **,** 22 **,** 26 **-** 36 **].** The figure shows the position of the amino acid changes associated with HFTC and/or HHS. The amino acid changes are placed in boxes corresponding to the observed phenotype; green box means HHS, blue box means HFTC and red box means the combined phenotype HFTC + HHS. Mutations placed above the figure are predicted to damage protein function, while missense mutations are placed below the figure. The orange box represents the transmembrane domain (TM, aa 20–37), the light red box represents the glycosyl transferase domain (aa 188–374), and the light blue box represents the ricin-B-lectin domain (aa 497–630). The shaded area represents the glycosyl transferase superfamily domain (aa 163–496).

Subject 1 was screened for mutations in all exons of *FGF23*, *GALNT3* and *KL*. No mutations were found in *FGF23* or *KL*, but a novel homozygous G-to T substitution in exon 3 of *GALNT3* (*c.767 G > T*) was identified, resulting in an amino acid change in position 256 (p.Gly256Val) (Figure 3). Subject 2 was also homozygous for this mutation, while her son and mother were heterozygous carriers. Subjects II-2, II-4, III-1, IV-1 and IV-2 did not carry the mutation. This substitution was not found in 192 healthy blood donors.

The detected mutation is not described in available databases and both the nucleotide and amino acid positions are highly conserved (phyloP: 5.86 and conserved up to C. elegans), and the variant is predicted as pathogenic by several prediction programs such as Polyphen2 [15], Align GVGD, MutationTaster and SIFT.

### Review of the literature

A summary of all articles describing HFTC and HHS in subjects with *GALNT3, FGF23 and KL* mutation is given in Additional file 1: Table S1.

In addition to the two subjects in this report, our search of the literature identified a total of 40 articles describing 54 affected subjects. This gives a total of 56 subjects (30 male; 26 female) from 35 different pedigrees. In 12 of the previously described cases of HFTC and one of the cases of HHS, we found that the cases had actually a combined phenotype of HFTC + HHS (“Phenotype revised” in Additional file 1: Table S1).

### Genotype and phenotype

*GALNT3* mutations were identified in 42 cases (22 men, 20 female; 75%) (Figure 3), *FGF23* mutations in 13 cases (8 male, 5 female; 23%), and a *KL* mutation was found in one female subject (2%). HFTC was the dominating phenotype in subjects with *GALNT3* mutations, whereas in subjects with *FGF23* mutation, HFTC and the combined HFTC + HHS phenotype were equally represented (Table 3). Interestingly, the isolated HHS phenotype was not found in subjects with *FGF23* mutation. Dental involvement was reported more often in subjects *GALNT3* than *FGF23* mutation (43% and 23%, respectively), whereas vascular calcification seemed more common with *FGF23* mutation than with *GALNT3* mutation (23% and 14%, respectively). Eye involvement was reported equally with both genotypes.

**Table 3:** **Review of the literature** [3,4,7,8,11,16-49]

**Phenotype**	**Total**	**Genotype**			**Sex** ^**1**^	
		***GALNT3***	***FGF23***	***KL***	**Male**	**Female**
	**N (%)**	**N (%)**	**N (%)**	**N (%)**	**N (%)**	**N (%)**
Total	56 (100)	42 (75)	13 (23)	1 (2)	30 (54)	25 (46)
Male	29 (54)	22 (52)	8 (64)	0 (0)		
Female	25 (46)	20 (48)	5 (36)	1 (100)		
HFTC	30 (54)	22 (52)	8 (62)	0	18 (60)	12 (48)
HHS	6 (11)	6 (14)	0	0	3 (10)	3 (12)
HFTC + HHS	20 (36)	14 (33)	5 (39)	1	9 (30)	10 (40)
Dental involvement	22 (39)	18 (43)	3 (23)	1	13 (43)	8 (32)
Vascular calcification	10 (18)	6 (14)	3 (23)	1	3 (10)	6 (24)
Eye involvement	9 (16)	7 (17)	2 (15)	0	6 (20)	3 (12)

^1^Between-sex comparison for *GALNT3* and *FGF23* mutation only.

### Sex and phenotype

Overall, males tend to have the phenotype of classic HFTC (60% of men), while females more often have manifestations of hyperostosis (HHS alone or HFTC + HHS; 56% of women) (Table 3). Vascular calcification was assessed in 18 cases, and was reported to occur more often in females than in males, whereas dental and eye involvement were reported more often in males (43% and 20% of the males and 32% and 12% of the females, respectively).

## Discussion

We describe a consanguineous Caucasian family with two affected siblings carrying a novel homozygous missense mutation in exon 3 of the *GALNT3* gene. The male has classic HFTC and the female had one episode of cutaneous nodules on her scalp in infancy and episodes of HHS in childhood.

Tumoral calcinosis is extremely rare in infancy; only 21 cases have been described in the literature [16,50-54], and of these only three previous cases have been associated with elevated serum phosphate levels [16,55,56]. The genetic mutations of previously described cases are unknown. Subject 2 had subcutaneous tumors on her scalp at the age of three weeks, which may have been the first manifestations of HFTC in this patient, with new tumors developing on her gluteal area at six years and thigh at seven years. There are examples of asymptomatic hyperphosphatemic children, who developed HFTC some years after hyperphosphatemia was first identified [57], and in one family, a small child, with homozygous mutation in *FGF23*, was hyperphosphatemic but asymptomatic, in contrast to her older sister with HFTC [17]. Our review of the literature shows that HFTC and HHS rarely manifested before the age of two years, with 78% of cases presenting between two and 13 years of age. The symptoms displayed in subject 2 in infancy may reflect *GALNT3* associated HFTC. This condition may be under-diagnosed in small children.

In our family, the clinical picture was complex and varied significantly with age. Band keratopathy, not previously reported in *GALNT3* associated HFTC or HHS, was found in the female at age 52. Eye involvement, with irritated, itchy eyes, has been present in both siblings since early childhood, but visual acuity has not been affected. There is only one previous report of band keratopathy associated with HFTC, but in that case the mutation was not known [58]. Reported eye manifestations in HFTC and HHS also includes calcifications on the eyelids, conjunctiva and the peripheral cornea [11,18-20] as well as angioid streaks of the retina [19,21,22]. Angioid streaks represent linear breaks in areas of calcification of the Bruch’s membrane separating the retina from the choroid, and may be complicated by retinal detachment are typically found in pseudoxanthoma elasticum, a disorder of ectopic calcification. Conjunctival and corneal calcification (CCC) is a well-known manifestation of metastatic calcification in end stage renal disease (ESRD) [59]. CCC occurs when the level of calcium and phosphate in tears approach their solubility product. As tears evaporate, and the fluid is concentrated, the result is deposition of calcium-phosphate salts on the corneal surface in the exposed interpalpebral region. It is most often located in the perilimbal region, and does not affect visual acuity. However, the most severe form, band keratopathy, can lead to visual impairment. In ESRD, the severity of CCC is positively correlated to the serum level of phosphate and the serum calcium × phosphate product, but not serum calcium levels [60,61]. CCC is also positively correlated to the occurrence of vascular calcification in ESRD [61]. The mechanisms of conjunctival and corneal calcifications are probably the same in HFTC and HHS, with a high calcium × phosphate product in serum and also other body fluids, such as tears. Our female subject is the first reported case of band keratopathy in *GALNT3* associated HFTC and HHS, and this outcome is probably the result of 50 years of untreated hyperphosphatemia.

Both our subjects have had severe dental involvement. From childhood their dental roots have been reported as abnormally short and bulbous, and from about 25 years age, their teeth started falling out despite good oral hygiene and regular dental care. Typical dental findings in HFTC and HHS are short, abnormal roots and obliteration of the root canals and pulp chambers. The lesions only partly resemble dentin dysplasia type I and II [23,24], and the specific dental lesion has been suggested as a phenotypic marker of HFTC [62].

The female had pain and stiffness in her hands and feet going on for several years, along with other diffuse symptoms, suggesting a rheumatologic disease. Thorough work-up failed to find an explanation, however, and the diagnosis of HFTC/HHS was not suspected until a calcified tumor arose in the male. This points to some important features of this disease: firstly, it is very rare, and even more so in Caucasians, and many clinicians will not be familiar with the symptoms and signs of the disease. This can lead to delayed diagnosis, as well as lack of recognition of complications of the disease. Second, in HFTC, there are often long symptom free intervals. This is most likely not associated with phosphate lowering treatment, but a feature of the disease [25].

In the first of her two pregnancies, the medical record describes pronounced placental calcifications. Placenta calcifications have not previously been reported in *GALNT3* associated HHS/HFTC. However, placental calcifications was also reported in a Caucasian female with HFTC [19] in whom genetic analysis later revealed mutation in *FGF23* [63]. Immunohistochemistry has shown a strong level of antibody staining for GalNAc-T3 in the Golgi apparatus and nuclei of normal human trophoblastic cells [64], and recent reports show evidence for the expression of the FGF receptor and its cofactor α-klotho in murine placenta [65]. These observations may support the hypothesis that HFTC/HHS may be associated with placenta calcifications. However, further studies are needed to examine the prevalence of placenta calcifications and possible placenta failure in human *GALNT3* and *FGF23* associated HFTC/HHS.

The female had age-inappropriate thyroid calcifications, but no clinical or biochemical symptoms or signs of thyroid dysfunction. This same feature has been described in one previous case of HFTC due to *GALNT3* mutation [26].

Both subjects had low levels of plasma intact FGF23 and elevated levels of c-terminal FGF23. This is in concordance with previous findings in HFTC and HHS, although in our cases the results deviated less from normal than in previously reported cases (Additional file 1: Table S1). *GALNT3* encodes the enzyme GalNacT3 responsible for O-linked glycosylation FGF23, thereby preventing the break down and inactivation of the functional full-length version of the protein [9]. Defective O-glycosylation of FGF23 due to *GALNT3* mutation, as well as mutation in the *FGF23* gene itself, will destabilize the FGF23 protein and lead to increased levels of FGF23 break down products. The level of c-terminal FGF23 will be increased, whereas the level of intact FGF23 will be low or inappropriately normal given the level of hyperphosphatemia. The explanation of the relatively less abnormal results for our two subjects is not clear, but could be due to sampling procedure, sample handling and transportation, or the different kits used for the analyses, as well as large inter-individual differences in FGF23 levels [66].

HFTC and HHS, previously described as separate entities, are now recognized as different manifestations of the same rare disease [4] of increased phosphate reabsorption from the kidney proximal tubuli. Some subjects display features of both phenotypes, whereas most have HFTC or HHS [27]. Some authors suggest a correlation between the type of mutation in the *GALNT3* gene and the phenotype [28]; most cases of homozygous missense mutations tend to have a HHS phenotype, while cases of homozygous nonsense mutations have a phenotype of HFTC. In our cases, however, the homozygous missense mutation has resulted in a HFTC phenotype in the male, and a combined HHS/HFTC phenotype in the female. Our review of all cases of HFTC and/or HHS caused by mutations in *GALNT3* showed that more cases than previously recognized had symptoms of HHS in addition to HFTC, and that HFTC seems to the dominating phenotype in males, while more females than males have manifestations of both HFTC and HHS. Our cases confirms the notion that there may be an underestimate of the prevalence of subjects with both phenotypes, as subject 2 in our material had symptoms of HFTC in infancy, and episodes of HHS in childhood, which remained unrecognized until the present study was conducted. The explanation for the great variation in phenotype, also within the same family, is not clear, and influence from different factors regulating calcification needs further clarification.

## Conclusion

We have described HFTC and HHS in a consanguineous Caucasian family with a novel *GALNT3* mutation, demonstrating new phenotypic features and significant variability in the natural course of the disease. A review of the literature shows that more subjects than previously recognized have a combined phenotype of HFTC and HHS. HHS and HFTC are two characteristic phenotypes in a spectrum of *GALNT3* mutation related calcification disorders, where the additional factors determining the phenotypic expression, are yet to be clarified.

## Additional file

Additional file 1: Table S1. Review of the literature.

## Abbreviations

iFGF23: Intact fibroblast growth factor 23
cFGF23: c-terminal fibroblast growth factor 23
TmP/GFR: Tubular maximum reabsorption of phosphate related to glomerular filtration rate
TRP: Tubular reabsorption of phosphate
